# Clinical and echocardiographic outcomes of combined CABG and MV repair

**DOI:** 10.6026/973206300212133

**Published:** 2025-07-31

**Authors:** Rajat Sindwani, Gurmeet Singh, Harneet Singh Khurana, Samir Kapoor, Vikrampal Singh, Sarju Ralhan, Rajesh Chand Arya, Rajiv Kumar Gupta

**Affiliations:** 1 Department of Cardiothoracic and Vascular Surgery, Hero Heart DMC Institute, Ludhiana, Punjab, India; 2 Department of Cardiothoracic and Vascular Surgery, All India Institute of Medical Sciences, Bathinda, Punjab, India; 3 Department of Anaesthesia, Dayanand Medical College and Hospital, Ludhiana, Punjab, India

**Keywords:** Effective regurgitant orifice area, New York Heart Association (NYHA), secondary mitral regurgitation, ischemic heart disease, coronary artery bypasses grafting

## Abstract

A mixed-method observational study evaluated patients undergoing Coronary Artery Bypass Grafting (CABG) with mitral valve (MV) repair for moderate to severe mitral regurgitation (MR). Most patients aged between 61-70 years (46.3%) and with 60% being male, 73% with Type 2 diabetes and 83.33% with triple-vessel coronary artery disease (CAD) (83.8%) and 15% with left main CAD. At discharge, 94.7% improved to NYHA class II and residual MR was eliminated in 97.1% patients. This study demonstrates significantly improved 12-month outcomes, with elimination of residual MR, supporting CABG with concomitant MV repair as a viable option for IMR patients despite high comorbidity.

## Background:

Primary Mitral regurgitation is degenerative valve-related heart disease associated with rheumatic, congenital, or aging-related etiology, which deteriorates the mitral valve (MV) function over time without showing any symptoms, whereas, ischemic mitral regurgitation (IMR) is secondary MV disease, originating from left ventricular (LV) remodelling in patients with chronic coronary artery disease (CAD) and/or ischemia, which causes pathologic changes in the MV structure and function [1, 2]. In comparison to LV remodelling, IMR has higher prevalence among individuals experiencing posterior myocardial infarction (MI) along with chronic CAD and the incidence of primary mitral regurgitation varies with age with prevalence of 60% among all MR patients, while secondary MR has a prevalence of about 65% among all mitral regurgitation patients, globally [3]. Secondary mitral regurgitation may develop due to dilatation and heart failure and its identification and diagnosis become a challenge, especially with the concurrence of rheumatic or degenerative aetiologies [4]. From the legendary works of Dr Alain Carpentier regarding mitral valve lesions, understanding the functional impairment is more important for treatment decision-making than the anatomical impairment IMR occurs due to increased tethering forces in the MV resulting from the displacement of papillary muscles, causing a migration of MV leaflets with their coaptation point apically and decreased LV contractility that causes reduction of tethering forces, perturbation of the systolic annular contractile motion, desynchrony of papillary muscles and intra-LV desynchrony [5]. Over time, significant improvements in the realm of surgical repair for chronic IMR have occurred, but the prognostic outlook of these patients remains controversial since regurgitation is only one facet of secondary MR and regurgitant flow through the MV has an unloading effect and reduces impedance in the LA-to-LV flow and the volume overload leads to further LV dilatation and increases the LV wall stress, which worsens LV performance [6]. The categorising and characterizing IMR is multifactorial and its severity varies according to the [5]. Essentially, identifying the surgical candidates for repair and replacement remains as the cornerstone of surgical secondary mitral regurgitation management to prevent not only adverse operative outcomes but also minimize replacement or repair failure rates [7, 8]. American Society of Echocardiography provides preliminary identification criteria for IMR, based on the presence of two conditions from: (1) the effective regurgitant orifice (ERO) area should be between 0.2 and 40 cm2, (2) width of *vena contracta* is 3-6 mm, (3) regurgitant jet (% area of LA) is 20-40% or above, (4) RV flow through is within 30-60 ml (Table 1 - see PDF). The supportive criteria include the LA chamber size, jet eccentricity, E-wave height and Doppler flow pattern of the pulmonary-vein (Table 1 - see PDF) [9, 10]. According to the 2020 ACC/AHA Guidelines for Valvular Heart Disease, surgical decisions should follow disease staging based on valve hemodynamics and LV anatomy such that ERO area is ≤0.4 cm^2^, regurgitant fraction <50%, regurgitant volume <60 mL and echo reveals regional wall motion abnormalities, reduced LV systolic function and LA dilation due to myocardial ischemia, a diagnosis of progressive IMR can be made-even in asymptomatic patients [11]. Moreover, the ACC/AHA guidelines suggest that 2D transthoracic echocardiography (TTE) may underestimate true ERO area in secondary MR because of the proximal semicircular convergence of MV leaflets above the chordae tendinea [10].

Among patients with prior MI or percutaneous coronary interventions (PCI), mitral regurgitation may lead to poor outcomes including deterioration of heart failure [12]. The coronary vasculature and myocardial perfusion should be assessed in chronic IMR patients and consequently, surgical replacement or transcatheter interventions are considered for patients with progressive or severe IMR, to prevent worsening HF due to non-treatment and no improvement with medical therapy [13]. MV repair thus can be performed during CABG with an operative mortality risk of 3-4% [14]. The presence of clinically significant residual mitral regurgitation introduces the need for a separate procedure of MV repair in the presence of patent grafts, which carries significant risk of peri-operative or 30-day mortality which can be avoided if pre-emptive MV repair is performed with CABG [15, 16]. Although CABG has remained as highly invasive procedure, but is preferred treat for CAD due to consistently high evidence of good prognosis [17]. The improvement with CABG in terms of survival rates remains debatable, but its role in significantly reducing the hospitalization rate has a considerable consensus [18]. Performing concomitant MV repair with CABG potentially rectifies the chronic IMR in many patients, whereas significant residual mitral regurgitation after CABG can worsen the prognosis, leading to late symptoms and decreased long-term survival [19]. Therefore, it is of interest to report in current research to investigate the postoperative and long-term clinical outcomes in patients with chronic CAD and IMR following concomitant MV repair and CABG.

## Patients and Methods:

## Study design and patient inclusion:

A mixed-methods observational study was performed that combined both prospective and retrospective study designs with aim to evaluate the clinical and echocardiographic outcomes of CABG with concomitant MV repair in IMR patients having underlying chronic CAD. Patients underwent preoperative angiography and their dyspnea symptoms were examined with the New York Heart Association (NYHA) questionnaire. Patients were included if they were aged above 18 years with CAD and moderate to severe IMR prescribed to undergo CABG with MV repair. Exclusion criteria were (i) aortic valve (AV) disease requiring replacement, (ii) organic MV lesions (rheumatic, infective, degenerative) and (iii) patients who needed surgical restoration of the left ventricle (LV). All research was performed in accordance with relevant guidelines/regulations and informed consent was obtained wherever required.

## Baseline clinical and echocardiographic evaluation:

All patients were assessed clinically for dyspnea symptoms with the NYHA functional class questionnaire and the patients underwent thorough preoperative echocardiography (transesophageal and transthoracic) for evaluation of regurgitation parameters (regurgitant fraction, regurgitant volume, *vena contracta*, LVESI, regurgitant orifice area and regurgitant jet %), LVEF and additional changes in ventricular diameters at systole and diastole. Only moderate and severe mitral regurgitation based on the ASE criteria listed in Table 1 (see PDF) were considered for the retrospective review and patient enrolment. Repeat echocardiography was performed on the postoperative day 5 or 7 and further follows up echocardiography conducted at 3 months, 6 months and 12 months, by same cardiac sonographer. The patients' medical histories and concomitant medications list were retrieved from the prescriptions and past surgery notes. The conditions of past illnesses were noted: diabetes, renal insufficiency, hypertension, previous CABG, previous PCI, heart failure, atrial fibrillation, or stroke.

## Surgical technique:

The procedure was started by inducing general anesthesia in each patient, based on the standard institutional protocol with invasive monitoring. After midline sternotomy and aortic cross clamping, cardiopulmonary bypass (CPB) was established using central aortic and bi-caval venous cannulation. Moderate hypothermia was maintained on CPB for CABG and after inducing hyperkalaemia cardiac arrest, MV repair was performed through trans-septal approach after opening right atrium. To reshape the mitral annulus, a 40-mm length mitral annulus band (SMB40; Sorin Biomedica SpA, Saluggia, Italy) was inserted from A1 to posterior annulus with further extension to A3 segment of mitral annulus, leaving only the A2 insertion free. With an identical surgical technique performed for all annuli and independent of annular size, the posterior annulus was shortened manually and brought forward, which reduced the mitral regurgitation [4, 14 and 15]. Intraoperative invasive monitoring included echocardiography along with CPB monitoring and other vital signs and the data were recorded at baseline along with details of old infarcts, number of diseased coronary arteries, age, gender, number of patent grafts and durations of CPB and aortic cross clamping (mins).

## Statistical analysis:

All study data were expressed as mean ± standard deviation Categorical data was expressed as numbers or frequencies with percentages *i.e.* n (%). For some variables, median values have been estimated. Data comparisons were analysed using Student's t-test for discrete data and ANOVA test for parametric data. Categorical data were compared using Chi-square (χ^2^) test when the expected frequency was ≤5. The differences in data between groups were considered statistically significant if the p-value was ≤0.05. All statistical calculations were performed using the Statistical Package for the Social Science SPSS version 21 software (SPSS Inc, IBM Corporation, Armonk, New York, United States).

## Results:

Baseline characteristics of the 80 patients were evaluated; 46.3% were aged between 61 and 70 years; observational cohort included more men (60%) than women. At baseline, severe IMR was present in 53.8% patients while 46.2% had moderate MR. The study group included patients with a remarkable level of comorbidities (68 patients with hypertension; 73 patients with type II diabetes; 15% patients with left main CAD). Triple vessel disease was quite commonly observed (83.8%) while 12 patients had double vessel disease (Table 2 - see PDF). The patients' NYHA classes had significantly improved at discharge as 94.7% of patients had NYHA class II dyspnea from the baseline levels (35% and 53.8% with NYHA class IV and class III, rest 11.3% with NYHA class II) (Figure 1 - see PDF). Over the course of 12 months' follow-up, the patients had maintained NYHA class I/II condition without deteriorating to class III/IV. The improvements in the NYHA functional class were significant (p=0.001). The LVESI is an important indicator of left ventricular remodelling and prognosis of ischemic myocardial disease. As observed in the follow-up echo conducted at discharge at intervals of 3, 6 and 12 months, the LVESI improved significantly over the 12 months' follow-up (LVESI improved from 61.42 ± 11.02 ml/m2 at baseline to 33.38 ± 4.38 ml/ m2 at 1-year follow-up (p value-0.000) (Figure 2 - see PDF). These results suggest that the chances of left ventricular modelling were reduced due to the surgical repair of mitral annulus in these patients with IMR despite their advanced CAD status. These improvements were complimented with enhancement in echo parameters including LVEF, which was 35.84±5.054% on admission signifying poor LV function that improved gradually to 42.59 ± 3.910% at 12-month follow-up showing a significant improvement (p value-0.001) (Figure 3 - see PDF). Regarding the postoperative mitral regurgitation grade, only 2.9% of the patients had residual mitral regurgitation at 12-month follow-up and 76 of 80 patients were discharged from the hospital with improved mitral regurgitation grades. No patients showed worsening of MR grade until 12-month follow-up. Together, the general physical condition of the survivors improved by a large extent.

The surgical technique lasted quite long, for more than 2 hours in some patients (mean CPB duration: 118.53 mins) while the aortic cross clamp time was short (mean 81.93 ± 34.81 mins). Ventilation support was required among the patients after withdrawing from the CPB support (mean duration of ventilation support: 18.78 hrs) and the overall perioperative experience was well managed without any perioperative deaths (Table 3 - see PDF). Several patients (72.5%) required preoperative IABP support due to loss of hemodynamic stability, all of whom were male. The average duration of hospital stay was 16.47 days. Four patients (5%) died during the first month with the primary cause of death being low cardiac output. For the rest 76 patients, survival was good up to 60 days post CABG+MV repair. Until the 12-month follow-up, 8 patients had died including one patient who died due to cardiac arrest. Along with the deaths, there were 34 cases of postoperative atrial fibrillation (42.5%), which were managed prior to exit from the operation theatre. Some unfortunate cases of cerebrovascular accidents had occurred (n=6 patients, 7.5%) following concomitant CABG and MV repair (Table 3 - see PDF). The 76 survivors were followed-up monthly, postoperative with 2-Dimensional Echocardiography being performed every month up to 12 months from the date of the index procedure. LVESI was monitored 7±12 months for left ventricular re-modelling and monitor the prognosis of ischemic myocardial disease. From the continuous follow-up of 76 patients who were discharged following MV repair with no postoperative residual mitral regurgitation and only 2.9% patients had mild mitral regurgitation at the 12-month follow-up, it was observed that 15% of population undergoing CABG were suffering from preoperative severe IMR (Table 4 - see PDF).

## Discussion:

IMR warrants focused research in cardiothoracic surgery due to its multifactorial CAD-dependent etiology and diverse treatment options, including PCI, MV replacement, or PCI with valvuloplasty for moderate to severe cases. An appropriate systolic leaflet coaptation depends on the synchronous anatomy and function of the MV annulus, leaflets, chordae, papillary muscles and the LV wall. Regurgitation occurs due to systolic retrograde flow from LV to LA because of inadequate leaflet coaptation during systole that generates a pressure gradient between LA and LV [6]. Distinguishing between primary mitral regurgitation due to organic disease and secondary mitral regurgitation is paramount to obtain good prognosis, considering that IMR has deleterious consequences of LV dysfunction with a worsening prognosis [20, 21]. From our study, key outcomes of CABG with MV repair were obtained as: (1) the surgical approach was highly effective for moderate to severe IMR instead of coronary revascularization, mitral valve replacement or revascularization combined with valvuloplasty (2) 97.1% patients who underwent MV surgery had no mitral regurgitation after 12 months. Chronic mitral regurgitation impedes the normal LA flow with relatively low compliance, resulting into high LA pressure, potentially leading to rapid pulmonary edema. Thus, mitral regurgitation can possibly occur within 1 week following an MI with (1) significant coronary obstruction (2) one or more LV segmental wall motion abnormalities and (3) significant structural disruption of normal MV leaflets and chordae tendinae and the prognosis worsens with chronic CAD [16]. The results wall motion abnormalities and LV remodelling leads to lateral and apical displacement of papillary muscles, which may be considered for papillary muscle approximation or percutaneous edge-to-edge repair. The major problem is the tethering MV leaflet having a posterior-medial scallop off the posterior leaflet (P-3) adjoining the commissural area and a neighbouring posterior infarction. Mitral annular dilatation occurs with leaflet tethering and the resulting dysfunction constitutes the usual ischemic mitral regurgitation type IIIb, with restricted systolic motion of the leaflet margin particularly during systole. Therefore, ischemic mitral regurgitation is combination of the effects of (a) a prior MI, (b) the MV leaflet tethering in the posterior-medial scallop and (c) type IIIb Carpentier dysfunction with restricted systolic MV leaflet motion [22, 23].

In their recent study, Guo *et al.* showed minimally invasive CABG to be safe and offering long-term survival among 566 patients as an alternative to sternotomy CABG [20]. The patients had exceptional long-term outcomes with only 4.5% patients reporting incisional pain (*i.e.* 19 patients) and the studied patients had NYHA Class II dyspnea or higher. Furthermore, >98% of treated patients had no pain at the sternotomy site. Aklog *et al.* investigated the outcomes of isolated CABG in 136 patients (mean age 70.5 years, 54% male) with NYHA class II symptoms with a mean LVEF 38.1% with a preoperative diagnosis of moderate IMR, without leaflet prolapse or pathologic changes [25]. Intraoperative TEE downgraded the severity of MR to mild or less (0 to 2+) in 34 of 38 patients. On postoperative TTE, 27 of 68 patients had moderate MR or higher (3 to 4+) (40%), 51% (35/68) experienced an improvement to mild mitral regurgitation (2+ type), while the mitral regurgitation resolved completely in only 9% (6/68) patients. The mean preoperative, intraoperative and postoperative MR grades were 3.06, 1.461 and 2.36, respectively (P<0.001) [25]. They concluded that for moderate IMR, CABG alone may cause residual mitral regurgitation, which may be diagnosed preoperatively that would be treated by concomitant CABG + mitral annuloplasty [21]. Malhotra *et al.* concluded that the LVEF is generally low in most secondary mitral regurgitation patients, which necessitates modern technique of MV repair with papillary muscle approximation that has been reported with better outcomes [26]. The patients with NYHA class III and IV heart failure must be operated under synchronous contraction that improves overall EF and led to significant reduction of wall motion abnormalities. Hence, even the significant level of residual MR recorded in this study does not affect the decreased long-term survival, but it may adversely affect long-term functional status and quality of life. Taken together, MV repair concomitant with CABG can be performed in patients with relatively low operative risk to improve the long-term functional status. This was particularly important in our patients, given the high incidence of preoperative LV dysfunction and congestive heart failure [26].

LVESVI has been used an indicator of LV remodelling and prognosis of IMR and following MV repair with CABG, the LVESVI improved significantly in our patients (Table 3 - see PDF). The significant improvement in NYHA class postoperatively up to 12 months was concordant with the results of Fattouch *et al.* who investigated the outcomes of isolated CABG or CABG with MV repair in 102 patients confirming significant improvement in postoperative LVEF following MV repair (p value-0.000) [27]. The improvements observed in this study are concordant with data from the RIME trial From their observations, Bax *et al.* concluded that CABG combined with annuloplasty led to LV reverse remodelling and significant reduction in LA size [17, 28]. The evaluation of the possibility of residual MR was important, since it repeats or creates residue after MV repair that may lead to backflow or impeded flow between the LA and LV. In particular, the most important source of residual MR could be suboptimal repair, insufficient downsizing of annuloplasty rings and incomplete revascularization. In our study, MR grades improved in all the patients with only 2.9% patients having residual MR at 12 months. The increased usage of IABP (72%) is similar to the observations of RIME Trial, in which the investigators reported the elective IABP insertion either before or at the end of surgery to optimise postoperative hemodynamic flows in high-risk patients.

Grossi *et al.* investigated the outcomes of CABG with reduction annuloplasty in 75 patients and reported 30-day mortality of 4.1%, which is lesser than that reported in this study (5%) [24]. In addition, one year mortality is nearly 15%. Although PASP is not an independent prognostic factor for evaluating MR outcomes, PASP ≥50 mmHg along with postoperative atrial fibrillation or LVEF ≤60% or LV end-systolic diameter ≥45 mm can predict worsening postoperative outcomes of MV repair irrespective of symptoms presenting or not. In this study, the mean PASP was restricted to 31.38 mmHg after discharge, despite the baseline values being a little high (mean, 45.57 mmHg). This could be considered as a factor for good recovery after MV repair and remodelling with survival and without complication. According to Lam and colleagues from the Cleveland Clinic, moderate IMR was associated with reduced survival as compared with CABG with matched bypass without moderate IMR (5-year survival, 73% vs 85%, p¼ » 0.003) [29]. The limitations of this study include some complex non-ischemic MR cases handled by surgeons with repair expertise, along with ischemic mitral regurgitation cases that are randomly allotted to different surgeons in our institution. In addition, a validated quality-of-life questionnaire was not administered to patients because of limited funding and dedicated personnel and early postoperative echocardiography was performed in all patients, but the echocardiograms were conducted based on the treating clinician's judgment and hence it can be conceived that some patients with mild to moderate MR may not have undergone echocardiographic assessment [9].

## Conclusion:

CABG modulated mitral valve repair for functional ischemic mitral regurgitation, predictably reduces mitral regurgitation and relieves symptoms. This operative strategy for the treatment of moderate to severe MR is associated with improved indices of ventricular geometry, improved NYHA functional class and excellent freedom from recurrent mitral insufficiency. Thus, patients with moderate to severe ischemic MR should undergo simultaneous valve repair at the time of CABG.

## Ethics approval:

The independent ethics committees of both hospitals provided approval; as retrospective data were reviewed at the Hero IDC Heart Institute (vide No. BFUHS/2K16p-TH/384), individual patient consent was taken wherever required. The Institutional Review Board of Dayanand Medical College provided approval of the study protocol prior to patient recruitment.

## Data availability statement:

The datasets used and/or analyzed during the current study are available from the corresponding author on reasonable request.

## Funding:

No funding was available for this study.

## References

[1] Deloche A (1990). J Thorac Cardiovasc Surg..

[2] Kataria R (2022). J Am Heart Assoc..

[3] Izquierdo-Gómez M.M (2018). e-J of Cardio Pract..

[4] Chehab O (2020). Heart..

[5] Carpentier A (1983). J Thorac Cardiovasc Surg..

[6] Cooley D.A (2010). Tex Heart Inst J..

[7] Koelling T.M (2002). Am Heart J..

[8] McCarthy P.M (2016). J Thorac Cardiovasc Surg..

[9] Di Mauro M (2006). Ann Thorac Surg..

[10] Fatima H (2020). J Cardiothorac Vasc Anesth..

[11] Otto C.M (2021). Circulation..

[12] Acker M.A (2015). N Engl J Med..

[13] Michler R.E (2016). N Engl J Med..

[14] Nappi F (2019). Ann Thorac Surg..

[15] Apostolakis E.E, Baikoussis NG. (2009). J Cardiothorac Surg..

[16] Nantsios A (2023). JTCVS Tech..

[17] Bax J.J (2004). Circulation..

[18] Dion R (1993). J Heart Valve Dis..

[19] Borger M.A (2006). Ann Thorac Surg..

[20] Guo M (2024). J Thorac Cardiovasc Surg..

[21] Nerlekar N (2016). Circ Cardiovasc Interv..

[22] Hadjadj S (2021). Can J Cardiol..

[23] Varma P.K (2017). Ann Card Anaesth..

[24] Grossi E.A (2011). J Thorac Cardiovasc Surg..

[25] Aklog L (2001). Circulation..

[26] Malhotra A.K (2017). J Cardiothorac Vasc Anesth..

[27] Fattouch K (2009). J Thorac Cardiovasc Surg..

[28] Granot Y (2025). Eur Heart J Cardiovasc Imaging..

[29] Lam B-Khanh (2005). Ann Thorac Surg..

